# Saturated fatty acids enhance daptomycin activity in a Fak-dependent manner in enterococci

**DOI:** 10.1099/mic.0.001751

**Published:** 2026-08-06

**Authors:** Sudan Maharjan, Troy A. Getty, Quiana Dickens, Amanda L. Hughes, Jillian Rundle, Elizabeth M. Fozo

**Affiliations:** 1 Department of Microbiology, University of Tennessee, Knoxville, Tennessee, USA

**Keywords:** cardiolipin, daptomycin, enterococcus, fatty acids, membrane fluidity, MIC

## Abstract

Antibiotic resistance of Gram-positive bacteria poses a major clinical challenge. Daptomycin, a lipopeptide antibiotic, is a treatment option for drug-resistant organisms. However, there have been clinical reports of daptomycin resistance and *in vitro* reports of daptomycin tolerance that can result in treatment failure. Daptomycin possesses a fatty acid tail that inserts into the membrane, and membrane fatty acid content can influence its binding. Given that, we examined the potential of fatty acids to enhance daptomycin activity against enterococcal strains. We demonstrate that the saturated fatty acids myristic and palmitic acids increase bacterial susceptibility to the antibiotic, even for a daptomycin-resistant clinical isolate. These effects were highly specific with no impact on daptomycin susceptibility of *Staphylococcus aureus*. Growth of enterococci with myristic or palmitic acid reduced membrane fluidity, potentially promoting tighter daptomycin insertion. However, we noted no difference in membrane permeability for cells treated with fatty acids and daptomycin. While cells exposed to saturated fatty acids experience greater membrane depolarization, the lack of consistent, significant time-dependent changes upon daptomycin treatment suggests that depolarization alone does not fully explain the increased sensitivity. A strain that is unable to incorporate exogenous fatty acids into phospholipids was insensitive to the effects of saturated fatty acids on its susceptibility to daptomycin, highlighting the necessity of fatty acid incorporation onto lipid headgroups for potentiation. Overall, our data suggest that the incorporation of specific saturated fatty acids within the enterococcal membrane likely disrupts lipid composition, membrane organization and cellular envelope homeostasis, ultimately contributing to enhanced daptomycin susceptibility.

## Introduction

Antibiotic resistance has been observed since they were employed for the treatment of bacterial infections, with resistance often reported shortly after introduction of the drug in the clinic [1–4]. The most recent comprehensive global data on bacterial antimicrobial resistance reported that such infections were directly responsible for ~1.27 million deaths worldwide and a secondary contributor to 4.95 million deaths [5, 6]. In the USA, over 2.8 million antimicrobial-resistant infections occur annually, resulting in more than 35,000 deaths [7]. The UK reported an estimated 66,730 serious antibiotic-resistant infections in 2023, exceeding pre-pandemic levels [8]. Gram-positive bacterial pathogens are major contributors to healthcare-associated infections and antimicrobial resistance [7, 9–12]. Indeed, infections caused by vancomycin-resistant enterococci (VRE) have remained elevated over the past several years, with little sign of reduction [7].

The standard clinical practice for treating Gram-positive bacterial infections is the penicillin-class antibiotics, including methicillin or vancomycin, a glycopeptide antibiotic [2, 9, 13–15]. Both target distinct aspects of cell wall synthesis, yet increasing resistance to these commonly used antibiotics has posed significant challenges to treatment strategies, particularly as the development of new antibiotics remains limited. Daptomycin, a lipopeptide antibiotic, is an alternative treatment for infections caused by resistant Gram-positive bacteria including VRE [12, 14, 16, 17]. Its antimicrobial activity arises from the insertion of its lipid tail into the bacterial cell membrane, leading to a reduction in membrane fluidity, alterations in the organization of membrane domains and subsequent disruption of membrane function and cell wall synthesis [15, 18]. There have been, though, reports of daptomycin-resistant species as well as continued struggles over the appropriate dosage for treatment [17, 19–22].

Alternative therapeutic strategies have been proposed for bacterial infections, including bacteriophage therapy, antimicrobial peptides, bacteriocins and immunotherapeutic antibodies [23–26]. Along with these, fatty acids with antimicrobial properties have gained attention as potential treatment options, either as standalone agents or in combination with antibiotics [23, 25, 26]. Fatty acids are often considered safer or less toxic than other antimicrobial agents, as many are naturally produced or ingested by the human body. For instance, sapienic acid (C_16:1 *cis* 6_), expelled on the skin surface, inhibits opportunistic *Staphylococcus aureus* [27]. A mouse wound infection model had improved recovery with the topical application of the fatty acid palmitoleic acid (C_16:1*cis*9_) [28]. It is, though, important to note that the sensitivity of an organism to a fatty acid can be species specific. As an example, *S. aureus*, but not *Enterococcus faecalis*, is sensitive to the eukaryotic-derived unsaturated fatty acids oleic and linoleic acids [29–33]. However, the situation is reversed for the saturated fatty acids myristic and palmitic acids which are detrimental to *E. faecalis* but not *S. aureus*.

Studies have shown that specific fatty acids can enhance the antibacterial activities of antibiotics when supplemented together as a co-treatment [23, 25, 34, 35]. There are also studies demonstrating that combining fatty acids with antibiotics can render inherently resistant bacteria sensitive to those antibiotics [25, 34, 36]. How fatty acids exert antibacterial activity is poorly understood [23, 25, 26, 36, 37]. It is presumed that the insertion of the fatty acid in the cell membrane disrupts the function of membrane proteins or compromises membrane integrity [25, 32, 36].

Both daptomycin and fatty acids exert their antibacterial effects by targeting the bacterial cell membrane and, for those cases studied, disrupt its function via altering membrane fluidity [15, 18, 29, 38, 39]. Given their similar effects, we hypothesized that fatty acids could enhance daptomycin susceptibility. Within, we demonstrate that the effectiveness of daptomycin against enterococcal strains was improved via the addition of specific saturated fatty acids, namely, myristic acid (C_14:0_) and palmitic acid (C_16:0_). We also report that the mechanism behind this potentiation is likely complex, as indicated through analyses of membrane fluidity, permeability and depolarization. Critically, the potentiation of daptomycin activity by fatty acids required the incorporation of the fatty acids onto lipid head groups and the production of the phospholipid cardiolipin. Thus, disruption of the phospholipid composition is a critical driver for this enhanced susceptibility.

## Methods

### Bacterial growth conditions

Bacterial strains used in this study are listed in Table S1 (available in the online Supplementary Material). Strains were grown statically in brain heart infusion (BHI; BD Difco) medium at 37 °C overnight. Overnight cultures were diluted to OD_600_=0.01 into BHI and grown statically at 37 °C. Fatty acids, lauric acid (C_12:0_), myristic acid (C_14:0_), palmitic acid (C_16:0_), stearic acid (C_18:0_), myristoleic acid (C_14:1*cis*9_) and palmitoleic acid (C_16:1*cis*9_) were added as indicated in the text. An equal final volume of solvent control (ethanol) was used as a control.

### MIC determination

The determination of the minimal inhibitory concentration, MICs, was via broth dilution with modifications [40, 41]. Overnight cultures were diluted as above into BHI supplemented with 1.5 mM calcium chloride (CaCl_2_) with the indicated fatty acids. BHI was chosen due to poor growth of enterococci strains in Mueller–Hinton and has been used previously [40]. Daptomycin was initially added to a final concentration of 0, 0.12, 0.25, 0.50, 1, 2, 4, 8 and 16 µg ml^−1^. The cultures were incubated for 24 h and observed for growth in each dilution tube to determine the MIC. Additional concentrations were used as warranted to obtain the final reported MICs. All experiments were a minimum of *n*=3; uninoculated media served as a negative control, while inoculated media lacking drug served as a positive control. For *E. faecalis* R712, there was notable debris and settling of the cultures; cultures were vortexed; then, the OD_600_ of the positive control (no drug or fatty acid added) versus test sample was determined, and the MIC was set to be at least 93% reduced growth compared to that of the positive control.

### Anisotropy

#### Simultaneous treatment of saturated fatty acids and daptomycin

Cellular fluidity via anisotropy measurements were performed using the fluorescent dye 1,6-diphenyl-1,3,5-hexatriene (DPH) [29, 38, 42]. Overnight cultures were diluted into BHI with 1.5 mM calcium chloride (CaCl_2_) and grown to early exponential phase; DPH was added at a final concentration of 7 µM and further incubated for 20 min at 37 °C. Each culture was then divided into four: one was treated with an equivalent amount of solvent control; the second was treated with 2 µg ml^−1^ of the indicated fatty acid; the third was treated with 2 µg ml^−1^ of daptomycin; and the fourth was treated with 2 µg ml^−1^ of the indicated fatty acid and 2 µg ml^−1^ of daptomycin. Cells were incubated for an additional 30 min and then harvested. Upon harvesting, cells were washed twice with 1× PBS via centrifugation at 2,739 ***g*** at 4 °C for 10 min. Bacterial pellets were resuspended in an equal volume of 1× PBS. DPH fluorescence was measured at 37 °C using an excitation wavelength of 350 nm and an emission wavelength of 428 nm on an Agilent Cary Eclipse Fluorescence Spectrophotometer using the Eclipse ADL software programme (Agilent Technologies). The steady-state anisotropy (*r*) was calculated using the equation by Lakowicz *et al*. for *n=*3 [43, 44]. Note that a lower *r* value indicates a more fluid membrane.

#### Daptomycin treatment of cells pre-grown with saturated fatty acids

Cells were grown at 37 °C in both the presence and absence of the indicated fatty acids to early exponential phase; DPH was added at a final concentration of 7 µM and further incubated for 30 min at 37 °C. An aliquot of cells was harvested and processed as above (time 0), and daptomycin was added to the remaining cultures. Cells were then harvested 15-, 30- and 60 min post-daptomycin addition following the above protocol.

### Sytox permeability measurement

Cellular permeabilization was assessed by monitoring the increase in fluorescence intensity resulting from the interaction of Sytox Green with cytoplasmic DNA as previously described with some modifications [37]. Bacterial cells were grown in 2 ml of BHI broth at 37 °C until they reached mid-log phase (OD_600_ ~0.3), in the presence and absence of different fatty acids as indicated. Cells were then centrifuged at 16,200 ***g*** for 4 min, and the supernatant was discarded. Cell pellets were washed twice with 8 ml of 1× Tris-HCl-CaCl_2_ buffer and centrifuged as above. The washed cells were subsequently resuspended in 2 ml of 1× Tris-HCl-CaCl_2_ buffer. Two hundred microlitres of the bacterial suspension was dispensed per well into a 96-well black plate with a flat, transparent bottom. Sytox Green was added at a final concentration of 0.1 µM, along with daptomycin at concentrations of 6 and 2 µg ml^−1^. Fluorescence measurements were recorded immediately using a BioTek Cytation 5 plate reader at an excitation wavelength of 504 nm and an emission wavelength of 524 nm, with readings taken at 5-min intervals for up to 2 h. As a positive control for permeabilization, cells were treated with 1% (v/v) Triton X-100. Shown are the changes in fluorescence over time for *n*=3 per condition/strain.

### Membrane depolarization assay

Cells were cultured overnight and diluted back to OD_600_=0.01 as described above in the presence of 1.5 mM CaCl₂ and incubated at 37 °C until OD_600_ ~ 0.3 in the presence of fatty acids (final concentration of 2 µg ml^−1^) as indicated in the text. Daptomycin (2 µg ml^−1^) was introduced into the culture, and an initial sample (time 0) was collected immediately. Subsequent aliquots were taken at 15, 30 and 60 min post-daptomycin addition.

For each time point, 200 μl of the culture was collected, washed twice with 2 ml of 1× PBS and centrifuged at 16,200 ***g*** for 5 min. The resulting cell pellets were resuspended in 1 ml of 1× PBS and stained with 10 µg ml^−1^ DiBAC_4_(3) (Life Technologies [32]). Staining was carried out in the dark at room temperature for 20 min, followed by another washing step as previously described. Cells were then fixed in 4% formaldehyde for 15 min in the dark, washed as above and resuspended in 1 ml of 1× PBS. Samples were diluted 1:10 before analysis using a CytoFLEX flow cytometer (Beckman Coulter), measuring fluorescence in the fluorescein isothiocyanate channel (excitation/emission: 490/516 nm). Differences in fluorescence intensity between treatment conditions were assessed using the geometric mean fluorescence intensity, obtained from triplicate measurements. Shown are averages±sd of *n*=3 biological replicates.

### Statistical analysis

All statistical analyses were done in GraphPad Prism Version 10. For each described assay, a minimum of *n*=3 biological replicates were used.

## Results

### Specific saturated fatty acids can reduce daptomycin MIC across enterococcal strains

Previous studies have demonstrated that host-derived unsaturated fatty acids could enhance stress tolerance to *E. faecalis* [29, 32, 33]. On the contrary, the addition of the saturated fatty acids myristic (C_14:0_) and palmitic (C_16:0_) acids resulted in poor growth and altered morphology of *E. faecalis* OG1RF [29, 33]. These fatty acids also reduced overall fluidity for *E. faecalis* [29]. Given that daptomycin can also decrease membrane fluidity, we hypothesized that the combination of myristic or palmitic acids could enhance daptomycin effectiveness not only within *E. faecalis* but also other Gram-positive bacteria [15, 18].

As past work with *E. faecalis* OG1RF demonstrated that a concentration of 5 µg ml^−1^ of either myristic or palmitic acid (22 and 19 µM, respectively) resulted in the selection of genetic suppressors, we opted to use a lower concentration to examine possible enhancement of daptomycin potency [32, 33]. We first confirmed that the addition of 2 µg ml^−1^ of either myristic or palmitic acid (8.8 and 7.8 µM, respectively) allowed for bacterial growth (Fig. S1). As shown in Table 1, with 2 µg ml^−1^ of either myristic or palmitic acid, the MIC of daptomycin was reduced for the tested enterococcal strains; however, this was not true for the *Staphylococcal* strains examined. *E. faecalis* OG1RF was particularly sensitized to daptomycin by the addition of myristic and palmitic acid with an ~85 and 75% reduction of drug needed to inhibit growth respectively, while the MICs for the other enterococci strains tested were reduced by ~50%.

**Table 1.** MIC of daptomycin (μg ml^−1^) in the presence or absence of saturated fatty acids*

Bacterial strains	MIC	MIC +5 µg ml^−1^ of lauric acid	MIC +2 µg ml^−1^ of myristic acid	MIC +2 µg ml^−1^ of palmitic acid	MIC +5 µg ml^−1^ of stearic acid
***E. faecalis* OG1RF**	6	2–4†	1	2	5
***E. faecalis* V583**	6	2–4†	3–4†	4	4
***E. faecalis* R712**	11	7	4‡	7	9
***E. faecium* TX0016**	6	2–4†	3–4†	2	3
***E. faecium* TX1330**	7–8	8	3–4†	4†	4†
***S. aureus* USA300**	4	4	4	3	4
***S. aureus* Newman**	1	2	2	2	2

*n=3.

†Welch’s t-test (*P*<0.05) in comparing the MIC with the indicated fatty acid to the MIC for the same strain with no fatty acid supplemented. No variation observed for the other conditions.

‡MIC with 2.5 µg ml−1 myristic acid for R712 was 2–4 µg ml−1 of daptomycin.

Previous studies indicated that the specific length of palmitic and myristic acids was the main driver of poor growth: the addition of the longer saturated fatty acid stearic acid was not as severe for growth [32, 33]. Supplementation with lauric acid (C_12:0_) had little impact overall on growth of *E. faecalis* OG1RF, but there were some observed morphological issues [33]. We decided to examine whether these fatty acids could also increase daptomycin susceptibility. The addition of lauric acid (5 µg ml^−1^, 25 µM) had little impact on growth [33], yet it did reduce susceptibility for the two *E. faecalis* strains and for *Enterococcus faecium* TX0016, but not *Enterococcus faecium* TX1330 (Table 1). For stearic acid (C_18:0_) addition (5 µg ml^−1^, 17.6 µM), there was increased susceptibility, though not as great as achieved with myristic or palmitic acids, ranging anywhere from a 17% decrease in drug for *E. faecalis* OG1RF to a 50% reduction in drug concentration needed to inhibit *E. faecium* TX0016 (Table 1).

Given that enhancement of daptomycin by saturated fatty acids was observed only with the enterococci strains, we focused our studies on those isolates.

### Unsaturated fatty acids with an equivalent carbon chain length do not reduce the daptomycin MIC

We wanted to conclude whether the reduced MICs observed with myristic acid (C_14:0_) or palmitic acid (C_16:0_) were specifically due to their saturation; thus, we examined the daptomycin MIC upon supplementation with the same length unsaturated fatty acids myristoleic acid (C_14:1*cis*9_) and palmitoleic acid (C_16:1*cis*9_). The addition of myristoleic acid had minor impacts on growth for most strains when supplied at 10 µg ml^−1^ (44.2 µM) (Fig. S2). Palmitoleic acid, as was reported previously, did impact growth when provided at 10 µg ml^−1^ (39.3 µM) for the enterococci strains; however, we did not observe selection of genetic suppressors (Fig. S2, data not shown [33]). As shown in Table 2, there was virtually no change in daptomycin MIC for strains supplied with myristoleic acid (C_14:1*cis*9_). The daptomycin MIC was also not impacted by palmitoleic acid presence for the two *E. faecalis* strains; however, for *E. faecium* TX0016, there was a threefold reduction in MIC when supplied with this fatty acid. Surprisingly, the addition of palmitoleic acid increased the MIC for *E. faecium* TX1330 – doubling the amount of drug needed to inhibit growth (Table 2).

**Table 2.** MIC of daptomycin (μg ml^−1^) for enterococcal strains with myristoleic acid or palmitoleic acid treatment*

Bacterial strain	MIC	MIC+10 µg ml^−1^ myristoleic acid	MIC+10 µg ml^−1^ palmitoleic acid
***E. faecalis* OG1RF**	6	6	7–8
***E. faecalis* V583**	6	6	7
***E. faecium* TX0016**	6	6	2
***E. faecium* TX1330**	7–8	8	16†

*n=3.

†Welch’s *t*-test (*P*<0.05) in comparing the MIC with the indicated fatty acid to the MIC for the same strain with no fatty acid supplemented. No variation observed for the other conditions.

### Simultaneous treatment of enterococci with saturated fatty acids and daptomycin has variable effects

Daptomycin, myristic and palmitic acid have been shown independently to decrease membrane fluidity in specific Gram-positive bacterial strains [15, 29, 38]. We hypothesized that the sensitization of enterococci strains to daptomycin by these fatty acids may be due to decreased membrane fluidity. We examined this via anisotropy using the dye DPH which is used as a proxy for membrane fluidity and rigidity [29, 38].

**Fig. 1.**
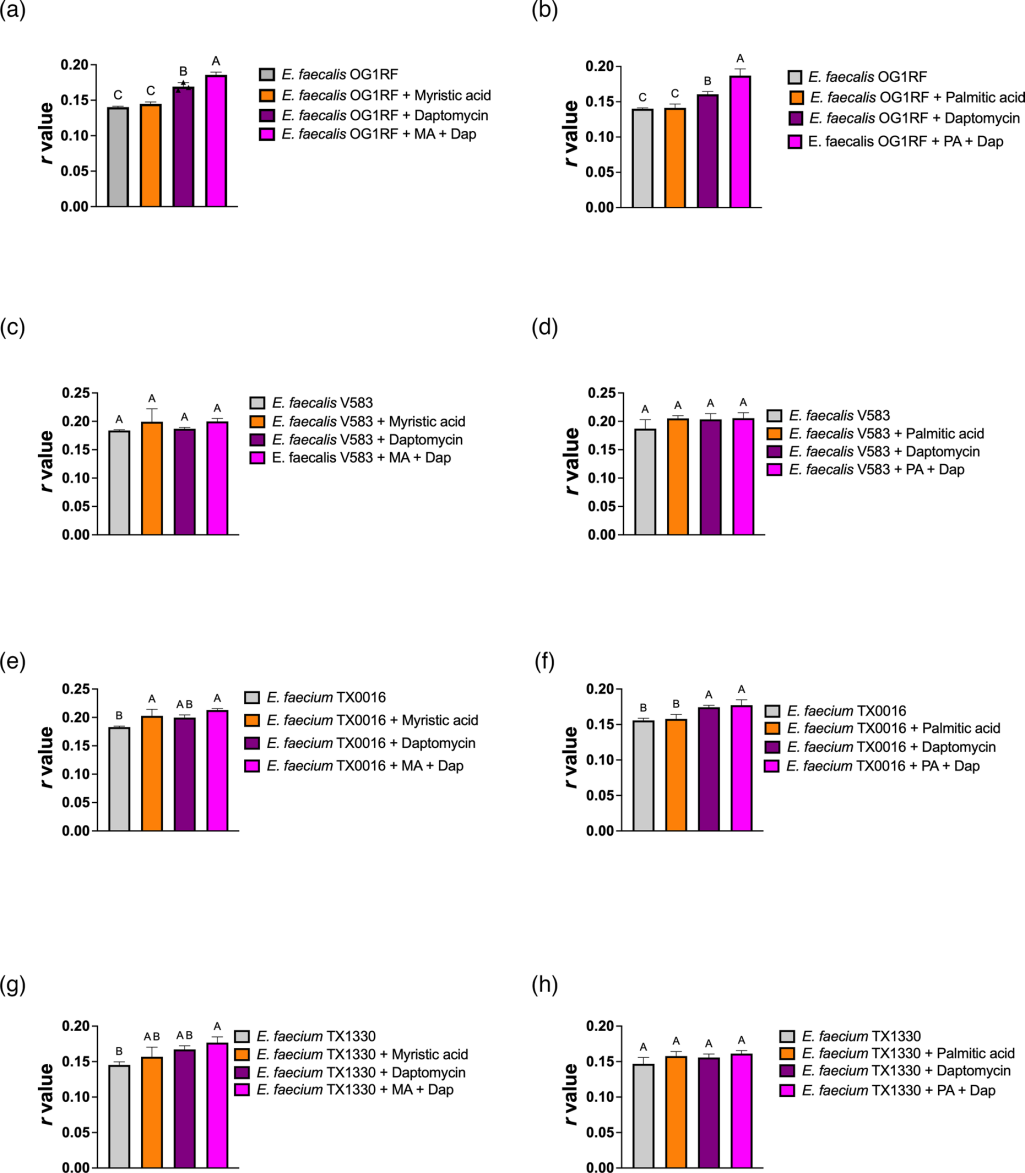
Membrane fluidity of enterococci is variable upon dual treatment with saturated fatty acids and daptomycin. Bacteria were grown to mid-exponential phase and cultures were split and treated with solvent control, 2 µg ml^−1^ of either myristic or palmitic acids, 2 µg ml^−1^ daptomycin or 2 µg ml^−1^ of the fatty acid and 2 µg ml^−1^ of daptomycin, as indicated for 30 min. Fluidity (via anisotropy) was then determined. Shown are the averages and sd of *n*=3. One-way ANOVA was used to determine *P*-values across conditions. Same letter above a column indicates *P*>0.05 (no statistical difference between those conditions with the same letter); different letters indicate *P*<0.05 (statistical difference between those conditions with different letters).

Exponential phase cultures were divided and treated either with solvent control, a saturated fatty acid, daptomycin or both, and fluidity was determined 30 min post-addition. We noted that the addition of 2 µg ml^−1^ of either saturated fatty acid did not alter fluidity (compared to solvent control) except when myristic acid was given to *E. faecium* TX0016 (Fig. 1). While daptomycin can increase membrane rigidity, we noted that this was strain dependent. For example, daptomycin treatment of *E. faecalis* OG1RF resulted in increased rigidity (higher *r* values), but there was no impact on *E. faecalis* V583. The combined effects of saturated fatty acid and daptomycin addition were also variable. For *E. faecalis* OG1RF, the dual treatment resulted in significantly greater membrane rigidity than any other treatment examined. For the other strains, either the dual treatment had no statistical difference compared to a monotreatment (i.e. *E. faecium* TX 00016 with daptomycin versus *E. faecium* TX0016 with both palmitic acid and daptomycin) or no changes in fluidity regardless of treatment condition (e.g. *E. faecalis* V583).

We hypothesized that cells pre-exposed to saturated fatty acids may be more sensitive to daptomycin-induced fluidity. Cells pre-grown with the saturated fatty acids were less fluid (higher *r* value) when compared to control cells prior to the addition of daptomycin (Fig. S3, time 0). This is similar to previous reports for *E. faecalis* OG1RF which showed reduced fluidity if the strain was grown in saturated fatty acids [29, 38]. The addition of daptomycin to fatty acid-grown *E. faecalis* cells resulted in a decrease in *r* value over time (decrease in membrane rigidity, Fig. S3). For *E. faecium* cells pre-grown with fatty acids, daptomycin addition had little to no impact on fluidity over time. However, for control cultures, daptomycin increased membrane rigidity (increased the *r* values), similar to what has been reported for other bacteria [15]. The final membrane fluidity observed after 60 min of daptomycin treatment for nearly all strains and growth treatments converged to nearly the same measurement, regardless of the presence of saturated fatty acids.

### Addition of daptomycin and saturated fatty acids has little impact on membrane permeability

When membrane fluidity decreases, phase separation may occur in which more fluid (i.e. those containing unsaturated fatty acids) lipids versus more rigid (i.e. those containing saturated fatty acids) segregate, resulting in a redistribution of proteins to the different domains [15, 45]. Over time, the membrane may weaken, resulting in ion leakage and eventual cell death. We therefore examined membrane permeability changes via a modified Sytox assay based on Sidders *et al*. to conclude whether the addition of saturated fatty acids resulted in increased membrane permeability upon daptomycin treatment [37].

**Fig. 2.**
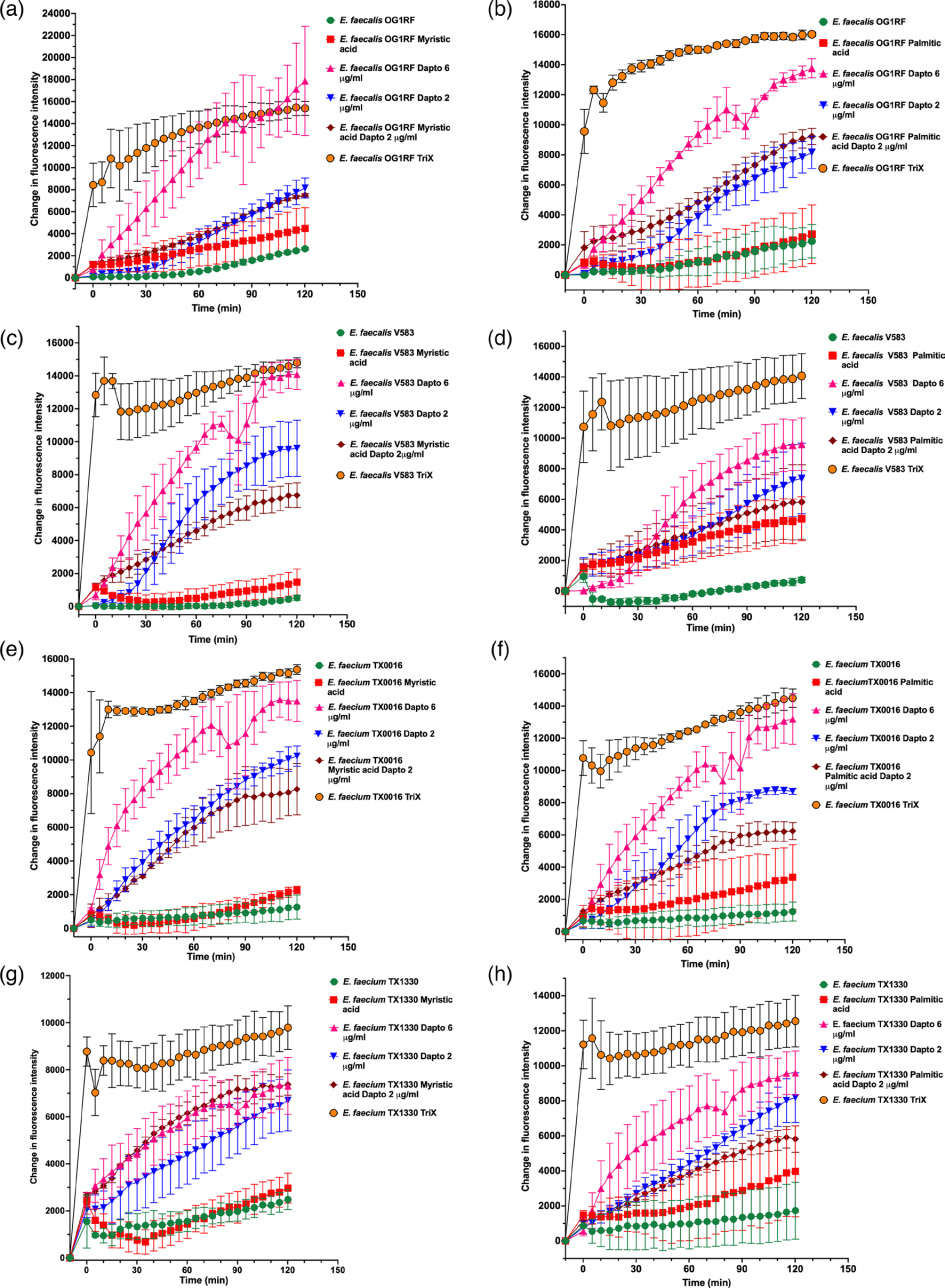
Saturated fatty acid supplementation does not permeabilize cells as well as high daptomycin treatment. Bacteria were supplemented as indicated with either 2 µg ml^−1^ myristic acid (**a, c, e, g**) or palmitic acid (**b, d, f, h**). Cells were treated with daptomycin at the concentrations indicated upon reaching exponential phase. Sytox green was used with an excitation wavelength of 504 nm and an emission wavelength of 523 nm. Significant changes (*P*<0.05) (not indicated here in fig) were observed in the first-hour time points for 2 µg ml^−1^ daptomycin (Dapto) treatment in both the presence and absence of each fatty acid (*n*=3).

As shown in Fig. 2, over time, regardless of treatment, there was an increase in permeability (increase in fluorescence), with the positive control (addition of 1% Triton X-100) producing the highest signal. For all strains, the addition of daptomycin at 6 µg ml^−1^ (approximate MIC) resulted in far higher permeability than the addition of fatty acids (2 µg ml^−1^) with the drug.

Given that early events upon daptomycin exposure could be driving fatty acid-induced sensitization, we opted to re-examine the data by focusing on the first 30 min of daptomycin exposure (Fig. S4). Still, within the first 30 min, treatment with 6 µg ml^−1^ daptomycin resulted in the largest changes in permeability for most strains and conditions. The addition of 2 µg ml^−1^ daptomycin to myristic acid-grown *E. faecalis* V583 and *E. faecium* TX1330 resulted in a similar increase in permeability within the same time span. For *E. faecalis* OG1RF cells treated with palmitic acid and daptomycin, there was an early increase in permeability similar to the high daptomycin treatment alone: this combination resulted in a greater change in permeability for *E. faecalis* V583 compared to 6 µg ml^−1^ daptomycin. Overall, though, permeability changes did not consistently correlate with the reduced MICs observed, suggesting additional mechanisms at play for sensitizing cells.

### Treatment with daptomycin on cells grown with saturated fatty acids had little impact on membrane depolarization

Daptomycin can cause membrane depolarization when given at high concentrations [18]. Further, when membrane fluidity is lowered, there can be a reduction in membrane potential [45]. Thus, we examined whether the combination of saturated fatty acids and daptomycin altered membrane depolarization using DiBAC_4_(3) (bis-(1,3-dibutylbarbituric acid) trimethine oxonol), a membrane potential-sensitive fluorescent dye [32].

Cells grown with a saturated fatty acid had a much higher basal level of DiBAC_4_(3) staining than cells grown without (*t*=0), except for *E. faecium* TX1600 grown with palmitic acid (Fig. S5). This was suggestive of pre-existing membrane potential changes, as was previously noted for *E. faecalis* OG1RF [32]. We noted that the difference in staining between the fatty acid culture and control was reduced over time in most cases upon daptomycin treatment.

**Fig. 3.**
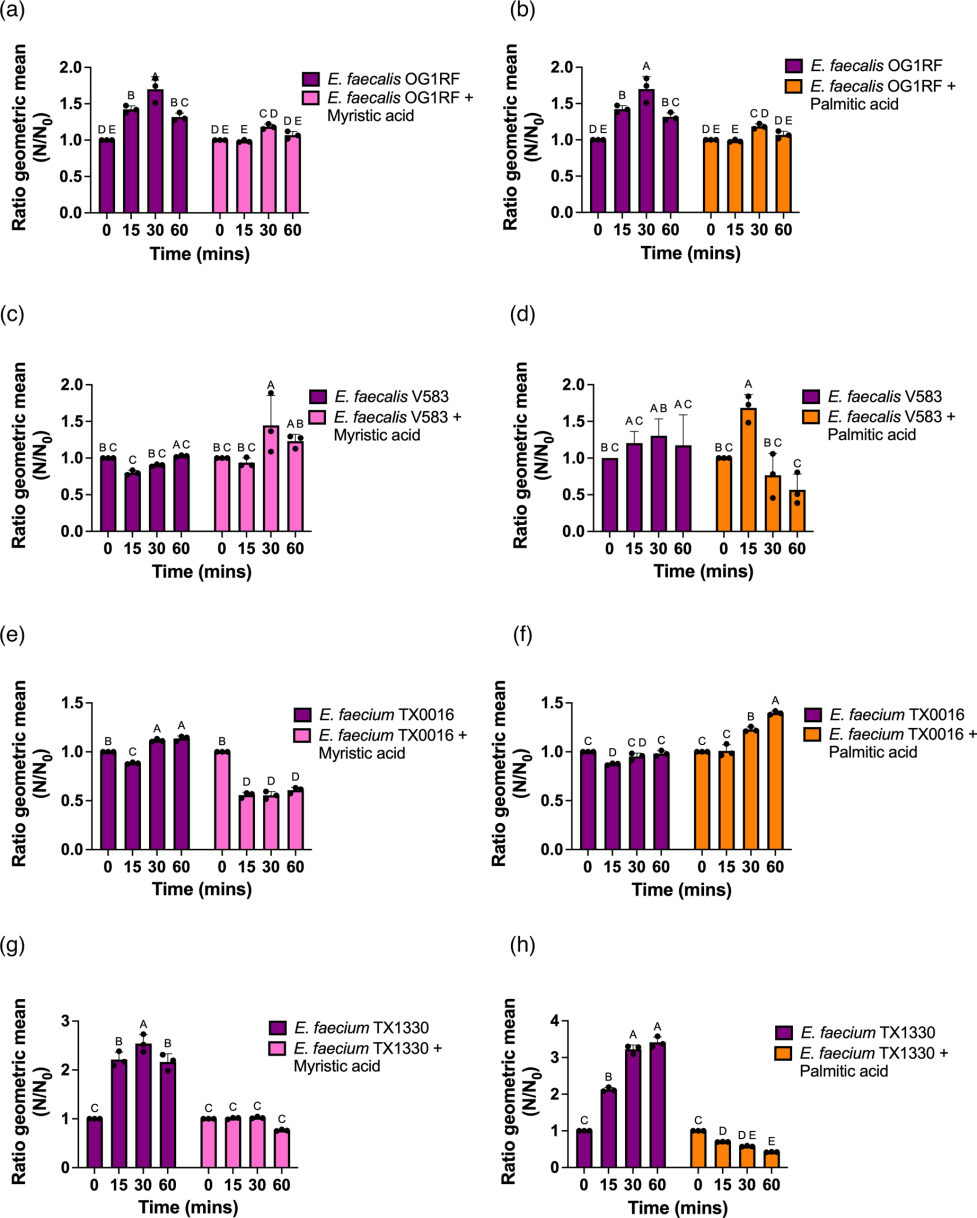
Membrane depolarization upon daptomycin is variable across strains and treatments. Bacteria were supplemented as indicated with either 2 µg ml^−1^ myristic acid (**a, c, e, g**) or palmitic acid (**b, d, f, h**). Daptomycin (2 µg ml^−1^) was introduced into the culture, and an initial sample (time 0) was collected immediately. Subsequent aliquots were taken at 15, 30 and 60 min post-daptomycin addition. Cells were stained with 10 µg ml^−1^ DiBAC_4_(3) and fixed with 4% formaldehyde. Shown are the averages and sd of *n*=3. Two-ANOVA with Tukey’s correction for multiple comparisons was used to determine *P*-values, same letter indicates *P*>0.05; different letters indicate *P*<0.05.

The addition of daptomycin to control cells had differential effects on DiBAC_4_(3) staining over time (Fig. 3). For example, treatment of *E. faecium* TX1330 with the antibiotic resulted in significantly more staining, but for other strains, there was more variation between time points and biological replicates. Cells exposed to either saturated fatty acid also had differential effects: *E. faecium* TX1330 exposed to myristic acid had no changes in depolarization over time, while when exposed to palmitic acid, there was an actual decrease in cellular depolarization over time. For *E. faecium* TX0016, the addition of daptomycin to myristic acid-grown cells resulted in a decrease in cellular depolarization over time, while daptomycin resulted in increased cellular depolarization over time. Again, there was a lack of consistent impacts on membrane depolarization with daptomycin sensitivity, indicative of a more complex mechanism.

### Saturated fatty acids can induce daptomycin sensitivity in a resistant clinical isolate but require incorporation of the fatty acid onto lipid headgroups

We also examined whether or not fatty acids could re-sensitize a daptomycin resistant isolate. *E. faecalis* R712 is a daptomycin-resistant clinical isolate recovered from a patient with bacteraemia [46]. Sequence and experimental evidence demonstrated that a mutation in the LiaFSR signalling system was the primary reason for the high drug resistance [47, 48]. Using this strain, we observed that the MIC to daptomycin was reduced by 64% with myristic acid and 37% by palmitic acid; increasing the myristic acid concentration to 2.5 µg ml^−1^ led to an even greater reduction by myristic acid (Table 1). Note that at these concentrations, cells were able to grow, albeit more slowly compared to the solvent control (Fig. S6).

*E. faecalis* OG1RF *ΔfakB 1,2,4,5/ΔtesE* strain (*Δquint* strain) does not encode the FakB proteins responsible for the incorporation of free fatty acids for lipid synthesis [38]. This strain accumulates exogenous fatty acids free within its membrane and is insensitive to the negative effects of saturated fatty acids given its inability to activate them for lipid synthesis [38]. We hypothesized that saturated fatty acids would have no impact on daptomycin sensitivity for the *Δquint* strain. Indeed, the MIC of daptomycin for the *Δquint* strain was equivalent regardless of the presence or absence of myristic or palmitic acids (Table 3). Thus, fatty acids can enhance daptomycin sensitivity if incorporated into lipids, not simply by accumulating as free fatty acids within the membrane.

**Table 3.** MIC of daptomycin for *E. faecalis* OG1RF Δquint* and *Δcls1/Δcls2*†

	MIC (μg ml^−1^)‡	MIC (μg ml^−1^) + 2 µg ml^−1^ of myristic acid‡	MIC (μg ml^−1^) + 2 µg ml^−1^ of palmitic acid‡
***E. faecalis Δquint***	3–4	4	3–4
***E. faecalis Δcls1/Δcls2***	0.5–2	1–2	1–2

*Strain lacks the *fakB* encoding genes and cannot incorporate exogenous fatty acids onto lipid headgroups [38].

†Strain lacking *cls1* and *cls2* encoding genes do not produce cardiolipin [39].

‡Minimum of *n=3*, *P* value >0.15 for fatty acid treatment versus control MIC (Welch’s t-test).

Daptomycin is known to cause alterations in cellular membrane organization, particularly impacting membrane domains of increased fluidity [15]. The phospholipid cardiolipin has been linked to daptomycin resistance and is considered to impart high fluidity within membranes [47, 49–51]. We examined whether saturated fatty acids could potentiate daptomycin sensitivity in a strain deleted for cardiolipin synthesis [39]. The MIC for an *E. faecalis* OG1RF *Δcls1/Δcls2* remained equivalent regardless of the presence of myristic or palmitic acids. Thus, overall membrane composition is critical for potentiation of daptomycin by saturated fatty acids.

## Discussion

Previous studies with *E. faecalis* OG1RF have shown that exposure to exogenous saturated fatty acids, specifically myristic and palmitic acids, impede bacterial growth significantly [29, 33]. Additional research reported that the combination of specific fatty acids with tobramycin (an aminoglycoside) can enhance antibiotic sensitivity against methicillin-resistant *S. aureus* (MRSA) strains [25]. Similarly, palmitoleic acid has been shown to potentiate vancomycin sensitivity in methicillin-sensitive *S. aureus* (MSSA), MRSA and vancomycin-resistant *S. aureus*, either lowering the required drug concentration or ‘resensitizing’ resistant strains [37].

Within, we demonstrated that low concentrations of myristic acid (2 µg ml^−1^ or 8.8 µM) or palmitic acid (2 µg ml^−1^ or 7.8 µM) enhanced daptomycin efficacy by lowering the MIC in enterococci (Table 1). These fatty acids, at the tested concentrations, did slow growth but not to the extent as palmitoleic acid, which only reduced daptomycin sensitivity in *E. faecium* (Figs S1 and S2, Table 2). Thus, these impacts are not likely due to a generalized slow-down of bacterial growth/metabolic activity. This potentiation effect of saturated fatty acids on daptomycin MIC was not seen in either *S. aureus* Newman (MSSA) or *S. aureus* USA 300 (MRSA) (Table 1). We particularly note that myristic and palmitic acids are toxic to the growth of *E. faecalis*, but not *S. aureus* [29–33]. Thus, the mechanism by which fatty acids enhance daptomycin activity is bacterial species and strain-specific, potentially influenced by differences in their membrane compositions or resistance mechanisms.

The enhancement of antibiotic activity by fatty acids has been linked to the disruption of cell membrane integrity through alterations in membrane fluidity, accumulation of membrane intermediates and dysfunctions in cell wall synthesis and division machinery, further compromising bacterial viability [23, 26, 37, 44]. Previous work has also demonstrated that saturated fatty acids reduce membrane fluidity for *E. faecalis* OG1RF, so we hypothesized that there would be a significant reduction in membrane fluidity which contributes to daptomycin potentiation [29, 38]. We also previously observed in *E. faecalis* OG1RF that the addition of myristic acid resulted in a far greater saturated: unsaturated fatty acid ratio compared to palmitic acid [32]. Some of this was driven by a decrease in *cis*-vaccenic acid in cells treated with myristic acid: *cis*-vaccenic acid is the dominant unsaturated fatty acid within the membrane of *E. faecalis*. While it is not clear why this fatty acid was so greatly impacted, *E. faecalis* can repress *de novo* fatty acid biosynthesis if supplied with exogenous fatty acids. However, these repressive effects are dependent on the fatty acid provided [52, 53].

Given the links to fluidity changes upon treatment of cells with either saturated fatty acids or daptomycin, we examined whether such changes were the primary driver for the heightened drug sensitivity when cells are given these fatty acids. In the strains tested, simultaneous treatment of saturated fatty acids and daptomycin had highly variable effects on cellular fluidity. For OG1RF, the dual-treatment did result in increased membrane rigidity compared to mono-treatment or even solvent control when using DPH as a proxy (Fig. 1). For the other strains, the trends were not clear. We did observe, however, that cells grown in fatty acid-supplemented media exhibited lower membrane fluidity (higher *r* value) compared to those grown in standard media before daptomycin exposure (Fig. S3). Interestingly, while a decrease in membrane fluidity was expected with the addition of daptomycin, in general, fluidity either remained constant or in some cases *increased* over time in fatty acid-treated strains upon daptomycin exposure. In contrast, either no change or a reduction in fluidity was observed in cells treated solely with daptomycin. These results suggest that the reduced membrane fluidity of cells pre-grown with myristic or palmitic acid may alter the interaction of daptomycin with the bacterial membrane, leading to differential effects on fluidity over time. Possible explanations for these findings include: either (i) the addition of daptomycin to fatty acid treated cells might have activated innate cellular mechanism(s) to compensate for the reduced membrane fluidity; or (ii) the disruption of membrane homeostasis caused by daptomycin’s interaction with phosphatidylglycerol (PG) may lead to localized PG depletion, triggering lipid redistribution that results in hyper-fluid regions of the membrane. This phase disorder and separation could damage membrane integrity and cause leakage [15, 18, 54, 55]. However, our permeability data counters this (Fig. 2).

Given that the fluidity and permeability analyses did not correlate well with the increased sensitivity of cells treated with fatty acids, membrane depolarization was investigated. At time zero, cells pre-exposed to saturated fatty acids had higher levels of depolarization than control cells as measured via DiBAC_4_(3) staining (Fig. S5). Yet, upon the addition of daptomycin, the response was varied across strains and fatty acid provided (Fig. 3). Indeed, for control cells, increases in depolarization over time were not consistently observed upon antibiotic addition. Thus, changes in membrane polarization upon daptomycin exposure do not completely explain the heightened daptomycin sensitivity upon exposure to saturated fatty acids.

Our recent studies demonstrate that for *E. faecalis* OG1RF, toxicity driven by myristic or palmitic acid is due to the incorporation into phospholipids: a *Δquint* strain, which lacks the necessary FakB proteins for activation of exogenous fatty acids for lipid synthesis, grows unimpeded at otherwise toxic concentrations of either fatty acid [38]. While the basal MIC to daptomycin of the *Δquint* strain was similar to that of the wild-type OG1RF, there was no change in MIC in the presence of either myristic or palmitic fatty acid (Table 3). Thus, a major driver for fatty acid potentiation of antibiotics requires the incorporation of saturated fatty acids onto phospholipids.

Why is the incorporation of these fatty acids needed for antibiotic potentiation? Data indicate daptomycin interacts with PG and potentially lipid II [18]. An increase in saturated fatty acids could impact either levels of PG or lipid II or their localization within the membrane. The subsequent effect(s) would then impact activity and/or localization of membrane proteins needed for energy and cell envelope synthesis: however, more in-depth studies are needed. Specifically, analyses examining the effects on membrane lipid composition, organization of fluid membrane domains, as well as the activity and precise localization of membrane-associated proteins, are warranted to fully elucidate the mechanism of increased daptomycin sensitivity.

Notably, the MIC of an *E. faecalis* strain deleted for its cardiolipin synthase genes was not impacted by the addition of either myristic or palmitic acid. Cardiolipin is considered to impart fluidity in the membrane given its structure and is thought to contribute to the formation of membrane domains [49, 51]. Data support that daptomycin resistant bacteria (including *E. faecalis* R712) have an altered distribution of the antibiotic in the cellular membrane compared to sensitive bacteria and that such resistant strains possess an altered membrane lipid composition [50, 56]. The lipid composition of *E. faecalis* OG1RF*Δcls1/Δcls2* strain is vastly different from the wild-type strain [39]. This loss of cardiolipin then likely drives an altered organization of membrane domains, which could contribute to the insensitivity of this strain to saturated fatty acid potentiation of daptomycin. Future studies are warranted to examine membrane domain formation across strains and treatment conditions.

A previous study found that fatty acids, such as oleic acid, could induce daptomycin tolerance in a manner independent of the LiaFSR signalling system in *E. faecalis* [57]. LiaFSR is a three-component system: LiaS is a sensor kinase that is activated upon cell envelope damage and then activates the response regulator LiaR. LiaF is a third component that inhibits signalling via LiaS, keeping the system tightly regulated. In the clinical isolate *E. faecalis* R712, a mutation in *liaF* allows for signalling through LiaS even under basal conditions, resulting in alterations in lipid organization, gene expression and, thus, daptomycin resistance [47, 48, 50]. Here, we note that saturated fatty acids can reduce daptomycin resistance even in this resistant isolate, supportive that toxic fatty acids are acting independent of the LiaFSR system (Table 1). Examination of the *E. faecalis* R712 genome (Biocyc.org) reveals four potential *fakB* (DegV domain encoding) genes and one potential *fakA* gene, similar to what has been described in *E. faecalis* OG1RF [58, 59]. Thus, the enhanced resistance is likely similar: incorporation onto lipid headgroups results in alterations in membrane architecture and potential membrane protein activity. However, this fatty acid reduction in daptomycin MIC was still above that of sensitive strains at the same concentration of drug, indicative that the LiaFSR system is still a primary driver of basal resistance in *E. faecalis*.

Within, we demonstrate that saturated fatty acids can serve as potential sensitizers of daptomycin for enterococci, even for resistant isolates. While the mechanism appears complex, this sensitization is dependent upon the Fak system and cardiolipin, indicating that fatty acid tail composition and placement, and likely membrane domain organization, contribute greatly to daptomycin sensitivity.

## Supplementary material

10.1099/mic.0.001751 Supplementary Material 1.

## Abbreviations

BHI: brain heart infusion
DPH: 1,6-diphenyl-1,3,5-hexatriene
MRSA: methicillin-resistant *Staphylococcus aureus*
MSSA: methicillin-sensitive *Staphylococcus aureus*
PG: phosphatidylglycerol
VRE: vancomycin-resistant enterococci
