# Supplementary material for: Saturated fatty acids enhance daptomycin activity in a Fak-dependent manner in enterococci
Source: Microbiology (Reading). 2026 Aug 6;172(8):001751. doi: 10.1099/mic.0.001751 (PMC13446751; doi:10.1099/mic.0.001751)
Supplement: Supplementary file 1 [file mic-172-01751-s001.pdf]

Supplemental data for:

Saturated fatty acids enhance daptomycin activity in a Fak-dependent manner in enterococci

Maharjan, Sudan*; Getty, Troy A.*; Dickens, Quiana; Hughes, Amanda L.; Rundle, Jillian; Fozo, Elizabeth M.#

*These authors contributed equally to this work

#To whom correspondence should be sent

Fozo, E.M. ORCiD: https://orcid.org/0000-0002-6928-2275

Affiliation(s)

Department of Microbiology, University of Tennessee, Knoxville, Tennessee, USA

Corresponding author and email address

Fozo, Elizabeth M.; efozo@utk.edu

Supplemental Data

Table S1. List of bacterial strains used in this study.

**Bacterial strains**	**Reference**
***Enterococcus faecalis* OG1RF**	Lab stock
Enterococcus faecalis* OG1RF *Δquint	Lab stock, (1)
Enterococcus faecalis* OG1RF *Δcls1/Δcls2	Lab stock, (2)
***Enterococcus faecalis* V583**	Lab stock
***Enterococcus faecalis* R712**	Generous gift from C. Arias, (3)
***Enterococcus faecium* TX1330**	Generous gift from B. Murray
***Enterococcus faecium* TX0016 (*Enterococcus faecium* DO)**	Generous gift from B. Murray
***Staphylococcus aureus* USA300**	ATCC
***Staphylococcus aureus* Newman**	Generous gift from A. Monteith

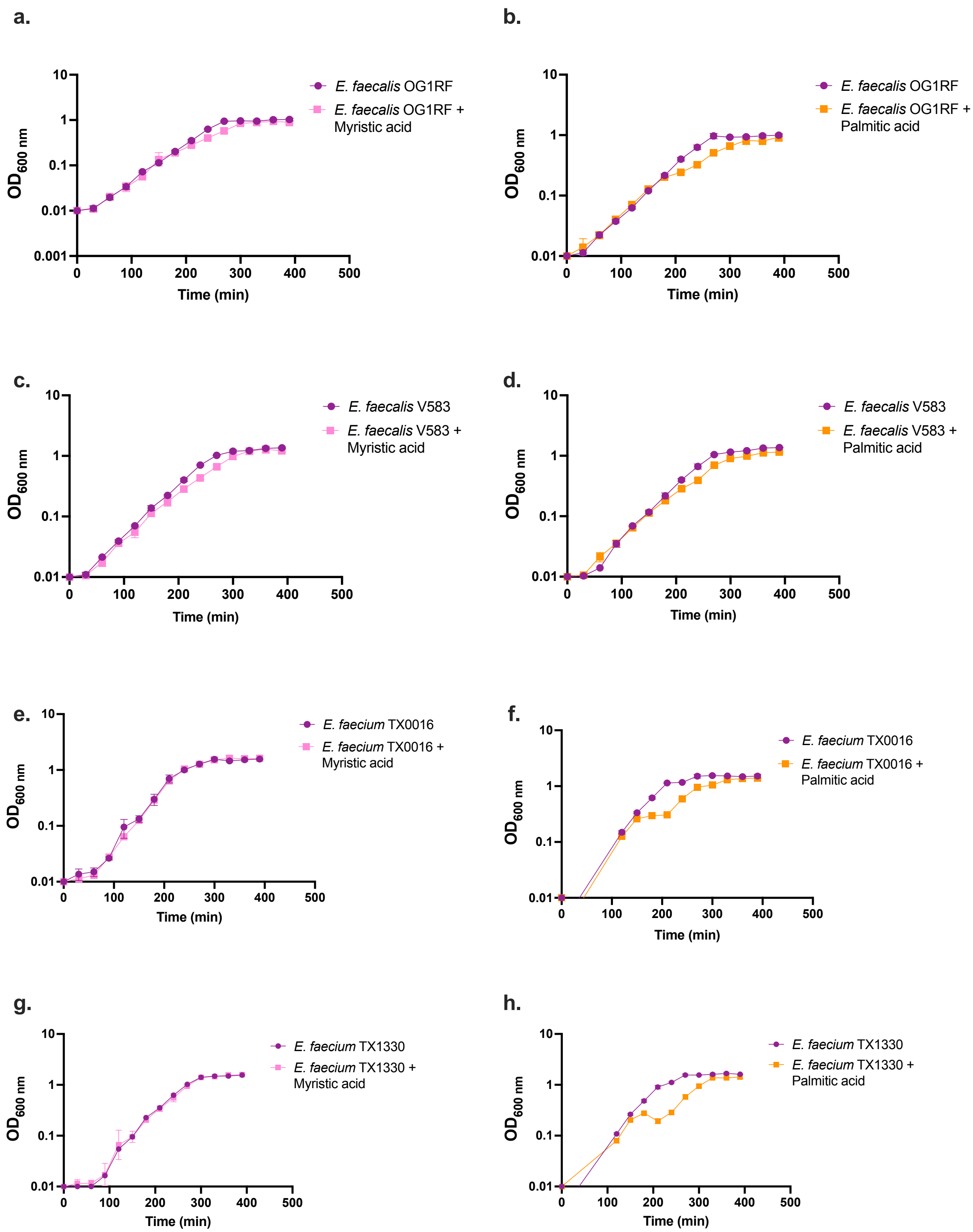

Fig. S1. Growth of enterococcal strains in myristic and palmitic acid. Bacteria were supplemented as indicated with either 2 µg/mL myristic acid (a, c, e, g) or palmitic acid (b, d, f, h). Shown are the averages and standard deviations for *n*=3.

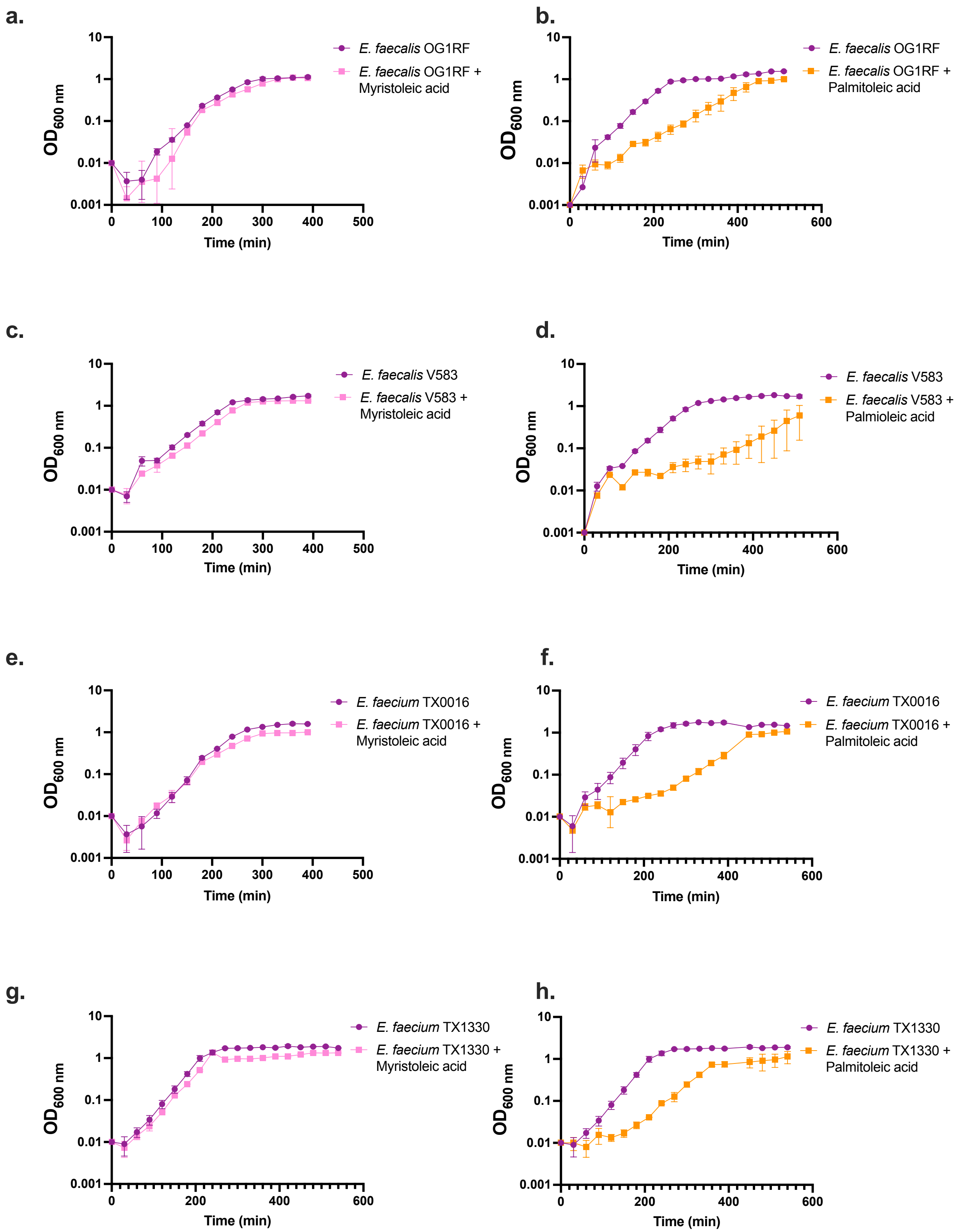

Fig. S2. Growth of enterococcal strains in myristoleic and palmitoleic acid. Bacteria were supplemented as indicated with either 10 µg/mL myristoleic acid (a, c, e,

g) or palmitoleic acid (b, d, f, h). Shown are the averages and standard deviations for *n*=3.

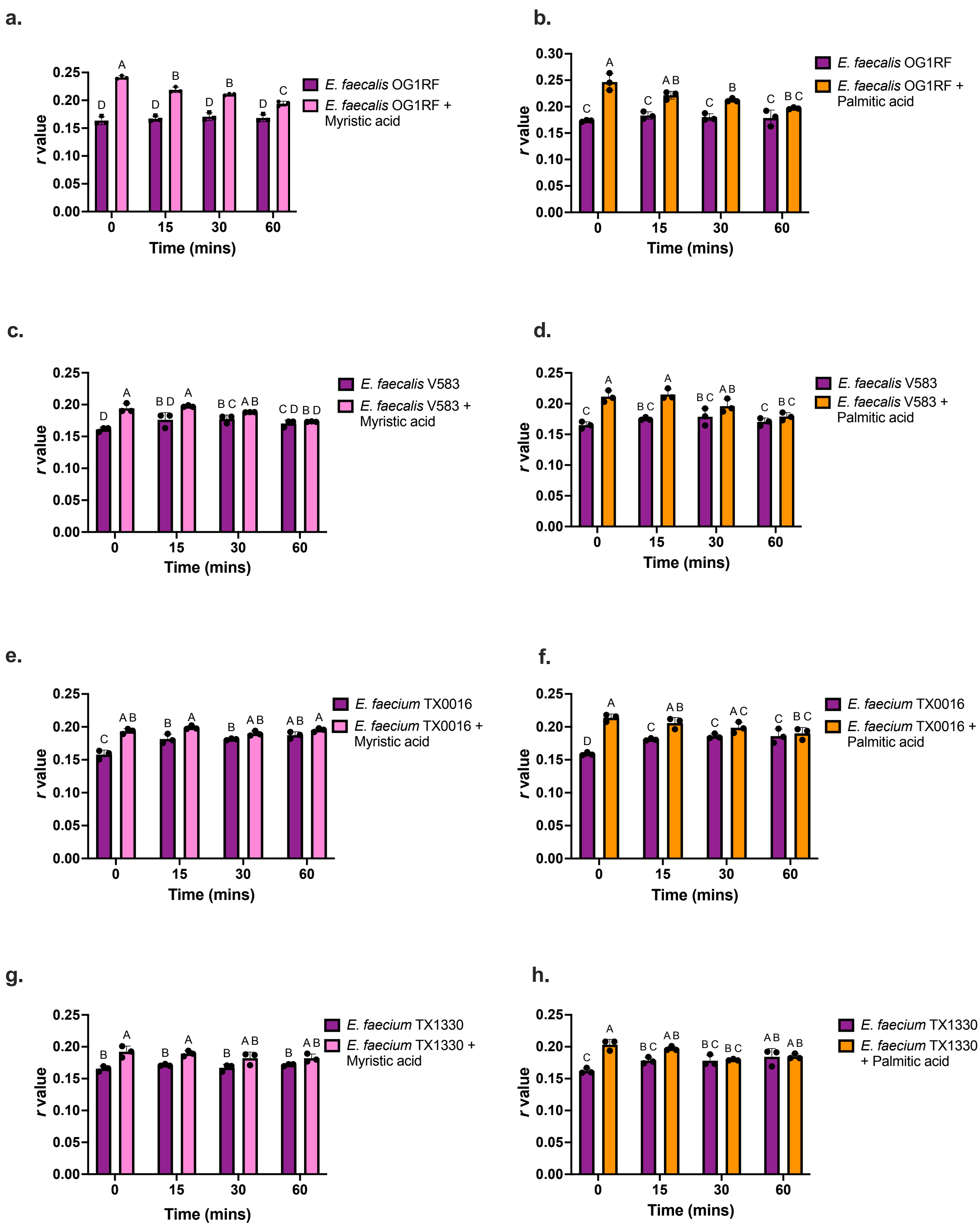

Fig. S3. Membrane fluidity of enterococci is variable upon daptomycin treatment. Bacteria were supplemented as indicated with either 2 µg/mL myristic acid (a, c, e, g) or palmitic acid (b, d, f, h) and fluidity (via anisotropy) of cells in exponential phase was determined following exposure to daptomycin (2 µg/mL). Shown are the averages and

standard deviations of *n*=3. Two-ANOVA with Tukey's correction for multiple comparisons was used to determine *p*-values. Same letter above a column indicates $P > 0.05$ (no statistical difference between those conditions with the same letter); different letters indicate $P < 0.05$ (statistical difference between those conditions with different letters).

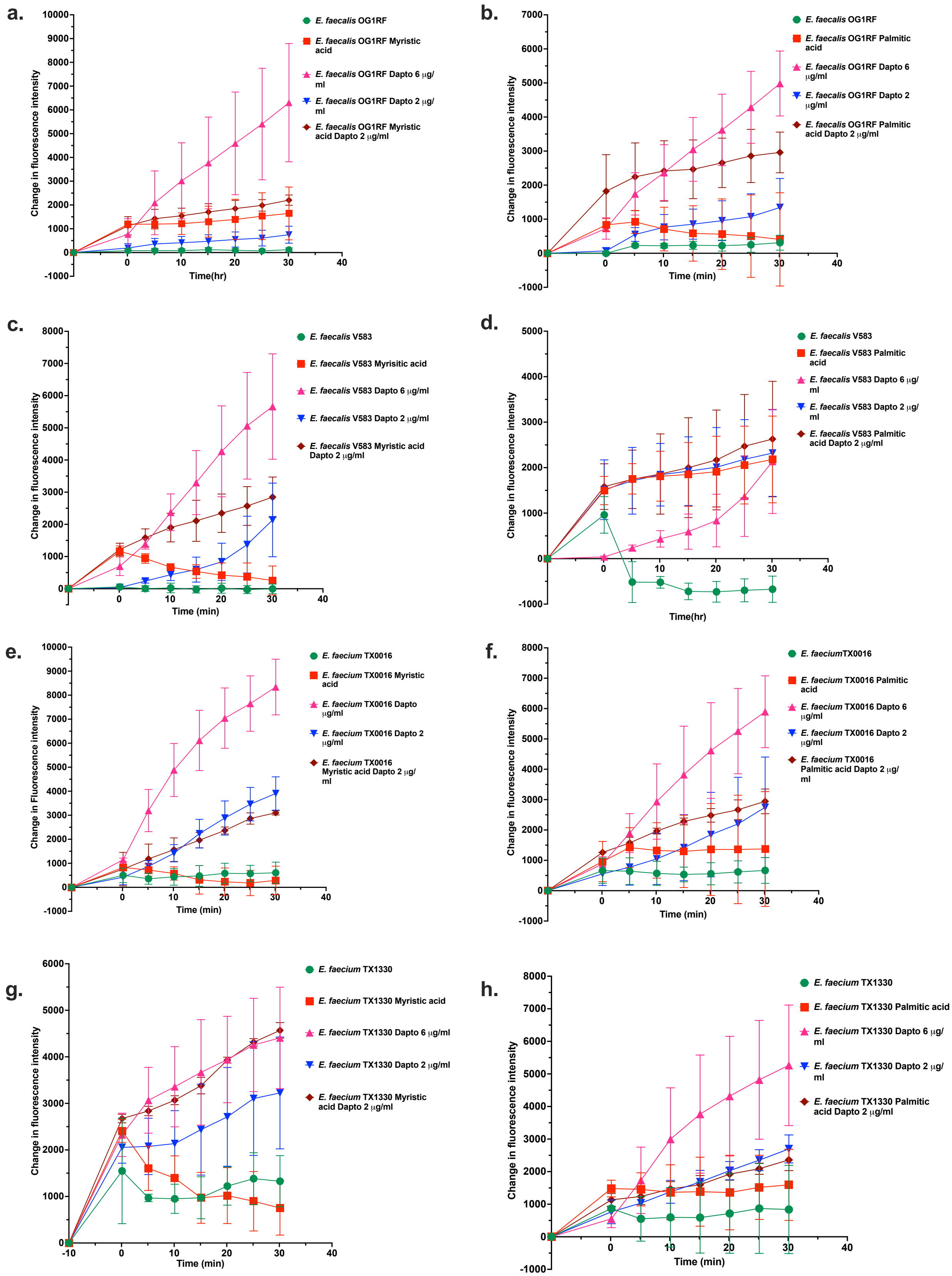
a.
Change in fluorescence intensity
Time(hr)
E. faecalis OG1RF
E. faecalis OG1RF Myristic acid
E. faecalis OG1RF Dapto 6 μg/ml
E. faecalis OG1RF Dapto 2 μg/ml
E. faecalis OG1RF Myristic acid Dapto 2 μg/ml
b.
Change in fluorescence intensity
Time (min)
E. faecalis OG1RF
E. faecalis OG1RF Palmitic acid
E. faecalis OG1RF Dapto 6 μg/ml
E. faecalis OG1RF Dapto 2 μg/ml
E. faecalis OG1RF Palmitic acid Dapto 2 μg/ml
c.
Change in fluorescence intensity
Time (min)
E. faecalis V583
E. faecalis V583 Myrisitic acid
E. faecalis V583 Dapto 6 μg/ml
E. faecalis V583 Dapto 2 μg/ml
E. faecalis V583 Myristic acid Dapto 2 μg/ml
d.
Change in fluorescence intensity
Time(hr)
E. faecalis V583
E. faecalis V583 Palmitic acid
E. faecalis V583 Dapto 6 μg/ml
E. faecalis V583 Dapto 2 μg/ml
E. faecalis V583 Palmitic acid Dapto 2 μg/ml
e.
Change in Fluorescence intensity
Time (min)
E. faecium TX0016
E. faecium TX0016 Myristic acid
E. faecium TX0016 Dapto μg/ml
E. faecium TX0016 Dapto 2 μg/ml
E. faecium TX0016 Myristic acid Dapto 2 μg/ml
f.
Change in fluorescence intensity
Time (min)
E. faeciumTX0016
E. faecium TX0016 Palmitic acid
E. faecium TX0016 Dapto 6 μg/ml
E. faecium TX0016 Dapto 2 μg/ml
E. faecium TX0016 Palmitic acid Dapto 2 μg/ml
g.
Change in fluorescence intensity
Time (min)
E. faecium TX1330
E. faecium TX1330 Myristic acid
E. faecium TX1330 Dapto 6 μg/ml
E. faecium TX1330 Dapto 2 μg/ml
E. faecium TX1330 Myristic acid Dapto 2 μg/ml
h.
Change in fluorescence intensity
Time (min)
E. faecium TX1330
E. faecium TX1330 Palmitic acid
E. faecium TX1330 Dapto 6 μg/ml
E. faecium TX1330 Dapto 2 μg/ml
E. faecium TX1330 Palmitic acid Dapto 2 μg/ml

Fig. S4: Daptomycin induced membrane permeability is treatment and strain variable at early time points. Bacteria were supplemented as indicated with either 2 µg/mL myristic acid (a, c, e, g) or palmitic acid (b, d, f, h)**.** Cells were treated with daptomycin at the concentrations indicated upon reaching exponential phase. Sytox green was used with an excitation wavelength of 504 nm and an emission wavelength of 523 nm. (*n*=3). Note, this is the same data presented in Fig.1.

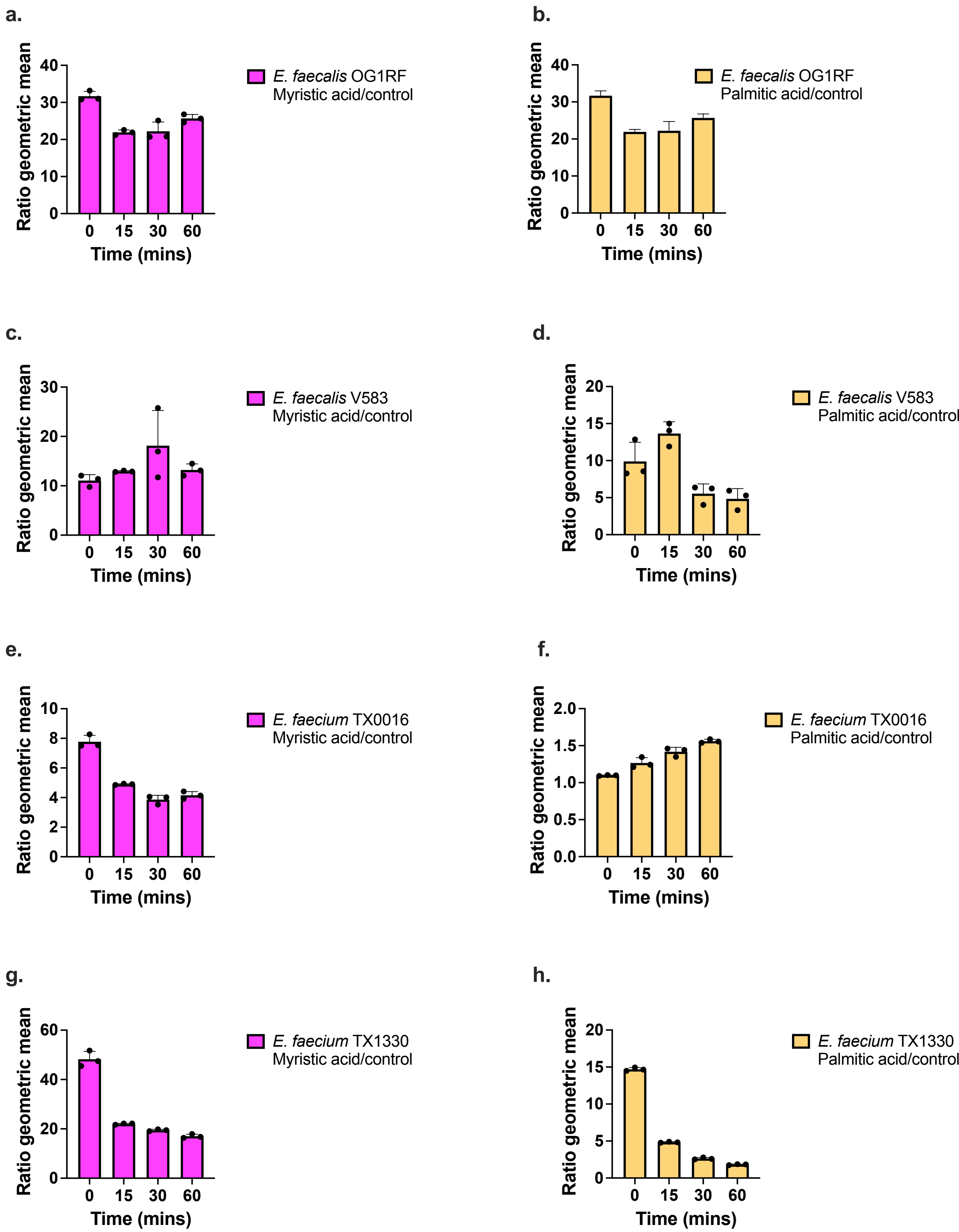

Fig. S5. Cells pre-grown with fatty acids experience greater depolarization than control cells. Cells were grown with no supplementation or with myristic or palmitic acid.

Daptomycin (2 μg/mL) was introduced into the culture, and an initial sample (time 0) was collected immediately. Subsequent aliquots were taken at 15, 30, and 60 minutes post-daptomycin addition. Cells were stained with 10 μg/mL $DiBAC_4(3)$ and fixed with 4% formaldehyde. Shown is the fold difference in geometric mean for cells pre-grown in fatty acids to their control cultures.

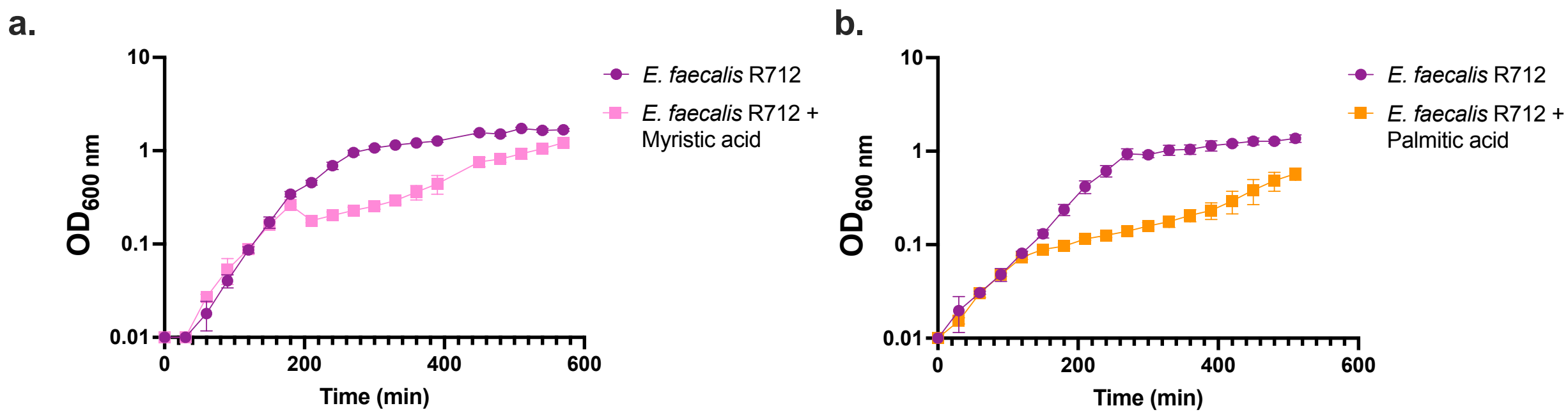

Fig. S6. Growth of *E. faecalis* R712 in myristic and palmitic acid. Strains were grown with final concentration of 2.5 µg/mL myristic acid (a) and 2 µg/mL palmitic acid (b). Shown are the averages and standard deviations for *n*=3.

REFERENCES

1. Johnston RD, Getty TA, Woodall BM, Maharjan S, Arnold NL, Seaton WB, et al. Unexpected contribution of the Fak system and the thioesterase TesE to the growth and membrane physiology of *Enterococcus faecalis*. J Bacteriol. 2025;207(7):e0012125.
2. Woodall BM, Harp JR, Brewer WT, Tague ED, Campagna SR, Fozo EM. *Enterococcus faecalis* Readily Adapts Membrane Phospholipid Composition to Environmental and Genetic Perturbation. Front Microbiol. 2021;12:616045.
3. Arias CA, Panesso D, McGrath DM, Qin X, Mojica MF, Miller C, et al. Genetic basis for in vivo daptomycin resistance in enterococci. The New England journal of medicine. 2011;365(10):892-900.
